# Cyclic distraction–compression promotes bone regeneration during distraction osteogenesis through the Piezo1–YAP–β-catenin axis

**DOI:** 10.3389/fbioe.2026.1772749

**Published:** 2026-05-26

**Authors:** Lian Tang, Lujun Jiang, Jialin Liu, Yufei Wu, Zi Wang, Juncheng Shen, Jinghong Yang, Chao Xian, Zhiyu Wan, Kai Liu, Yanshi Liu, Zhong Li

**Affiliations:** 1 Department of Orthopedics, Affiliated Hospital of Southwest Medical University, Lu Zhou, China; 2 Sichuan Provincial Laboratory of Orthopaedic Engineering, Lu Zhou, China; 3 Stem Cell Immunity and Regeneration Key Laboratory of Luzhou, Lu Zhou, China; 4 Department of Oral Implantology, The Affiliated Stomatological Hospital, Southwest Medical University, Lu Zhou, China; 5 Luzhou Key Laboratory of Oral & Maxillofacial Reconstruction and Regeneration, Lu Zhou, China; 6 Southwest Medical University, Lu Zhou, China; 7 Department of Orthopedics, Chongqing Medical University, Chongqing, China

**Keywords:** accordion technique, cdc, cyclic distraction–compression, distraction osteogenesis, do, Piezo1

## Abstract

**Background:**

Distraction osteogenesis (DO) is an effective reconstructive strategy for large bone defects, but its clinical application is constrained by slow regenerate maturation and prolonged consolidation. The cyclic distraction–compression (CDC, accordion technique) has shown promise in enhancing bone formation; however, its underlying mechanotransduction mechanisms remain poorly understood.

**Methods:**

A rat femoral DO model was established and CDC was applied during the mid-consolidation phase. To interrogate mechanistic pathways, a Piezo1 agonist (Yoda1) or inhibitor (GsMTx4) was locally administered. Bone regeneration was evaluated by serial radiography, micro-computed tomography, biomechanical testing, histomorphology, and immunohistochemical analyses.

**Results:**

CDC significantly enhanced regenerate bone volume, mineral density, structural continuity, and mechanical properties compared with controls. Activation of Piezo1 further amplified these effects, whereas Piezo1 inhibition attenuated CDC-induced improvements. At the molecular level, CDC increased the expression of Piezo1, YAP, and β-catenin, accompanied by elevated osteogenic markers (RUNX2, OPN) and angiogenic markers (VEGF, CD31). These findings indicate that CDC promotes coupled osteogenesis and angiogenesis within the distraction regenerate through Piezo1-dependent signaling.

**Conclusion:**

Our study suggests that CDC accelerates bone regeneration during distraction osteogenesis, potentially through Piezo1–YAP–β-catenin–associated signaling and enhanced osteogenic–angiogenic coupling. These results provide mechanistic insight into the accordion technique and support the development of optimized mechanical and mechanosensory-targeted strategies to improve DO outcomes.

## Introduction

The reconstruction of critical-sized segmental bone defects in the long bones presents a formidable challenge in orthopedic surgery. Inadequate management often precipitates severe complications, including nonunion, chronic infection, or permanent functional disability (Filippo et al., 2021; Erika et al., 2017; Johannes et al., 2009; Enrique and Enrique, 2017). Current therapeutic modalities, such as autologous bone grafting, allograft transplantation, and the Masquelet induced membrane technique, constitute the standard of care. However, each modality is associated with inherent limitations, including donor site morbidity, limited graft availability, and immune rejection (Magdy et al., 2018; Graci et al., 2010; Matheus Lemos et al., 2019; Po‐Kuei et al., 2018; Gunasekaran and Badri, 2013a; Henry et al., 2005).

Distraction osteogenesis (DO) is an effective method for promoting new bone regeneration by gradually creating new bone callus through the distraction of bone segments. Since its introduction by Ilizarov, DO has been widely applied globally for correcting bone deformities and repairing bone defects, particularly in the fields of oral and maxillofacial surgery, orthopedics, and trauma orthopedics (Ilizarov et al., 1982; Gunasekaran and Badri, 2013b; В Г et al., 2013; Ю Б et al., 2020; Barakat et al., 2020; Ю Б, 2012; Jan and Daniel, 2017; Munetomo et al., 2013). Its advantages include inducing endogenous bone formation, reducing graft-related complications, and stimulating surrounding soft tissue regeneration, making it a superior bone tissue engineering technique (Steve et al., 2014; Saw et al., 2019; Doron and Mark, 2017; Barakat et al., 2008; Asim and Reggie, 2013). However, DO suffers from slow new bone formation and a relatively high incidence of complications. Therefore, improving its efficiency to reduce patient burden is urgently needed (Carlo et al., 2020; Ulrich et al., 2013; Dror, 2016).

In recent years, to accelerate new bone regeneration in DO, researchers have explored methods such as bone grafting, ultrasound or electromagnetic field stimulation, pharmaceutical interventions, cell therapies, and vascular regeneration stimulation. While some success has been achieved, most approaches remain in the preclinical stage with limited practical application (Yueliang et al., 2021; Mohammad et al., 2017; Yohei et al., 2020; Aaron et al., 2014; Qi et al., 2021; Yon et al., 2020; Feng et al., 2021a; Feng et al., 2021b). Therefore, identifying cost-effective methods to promote new bone formation in distraction sites has become an urgent clinical challenge requiring resolution.

Cyclic distraction–compression technique (CDC), also known as the Accordion Technique (AT), stimulates bone regeneration through alternating tensile and compressive stresses. This drug-free, simple, and non-invasive method creates an ideal biomechanical environment to activate the patient’s osteogenic potential. It has been preliminarily applied in clinical settings with promising outcomes (Gavriil, 1990; Qun et al., 2017; Asim et al., 2015; Mohammed Anter et al., 2021; Muhar et al., 2005). However, the specific mechanisms by which CDC promotes new bone regeneration require further investigation.

New bone formation during DO is closely associated with angiogenesis. Studies indicate that during bone tissue development, both endochondral and endomembranous ossification are accompanied by vascular formation (Gérard et al., 2009). Hypoxia during DO is generally recognized as a key factor activating the osteogenesis-angiogenesis coupling. For instance, by establishing a hypoxic environment, HIF-1α activation promotes VEGF expression, thereby stimulating angiogenesis and osteogenesis (Kai and Björn, 2016; Yanshi et al., 2022a). The Notch signaling pathway also plays an active role in both osteogenesis and angiogenesis, particularly by establishing the YAP/TAZ-Notch circuit through the upregulation of YAP and TAZ, thereby promoting the coupling of angiogenesis and osteogenesis (Feng et al., 2024). However, the molecular mechanisms underlying the osteoangiogenic coupling remain incompletely understood, especially regarding its role in AT. Exploring this direction may further elucidate the mechanisms of CDC technology.

Piezo1 is a mechanosensitive cation channel that detects physical forces and converts them into electrical signals, playing a crucial role in various biological processes including the maintenance of bone homeostasis (Sanjeev et al., 2015; Bertrand et al., 2010; Jason et al., 2016). YAP and TAZ are key transcription coactivators in the Hippo signaling pathway, whose activity is regulated by both mechanical and biochemical signals (Sirio et al., 2011). Research indicates that the YAP/TAZ axis promotes osteoblast differentiation by activating Runx2 (Zhen et al., 2021). β-catenin, an intracellular protein involved in the Wnt signaling pathway, plays a role in promoting osteoblast development and proliferation (Yaoqin et al., 2001; Hongliang et al., 2004). Research indicates that Piezo1 may act through the Wnt/β-catenin pathway. Upon activation, Piezo1 promotes calcium ion influx, leading to YAP dephosphorylation and nuclear translocation. There, YAP binds to β-catenin to form the YAP/β-catenin complex, which subsequently upregulates osteogenic and angiogenic factors to promote bone regeneration (Yuanyuan et al., 2022; Yunlu et al., 2022).

Research has revealed that the Piezo1–YAP–β-catenin signaling axis plays a crucial role in regulating bone homeostasis. Zhou et al. first demonstrated the key role of Piezo1/2 synergistically activating the NFAT-YAP1-β-catenin pathway in bone formation (Taifeng et al., 2020). Through studies on periosteal stem cells, Liu et al. further showed that this axis also plays a critical role in fracture healing (Yunlu et al., 2022). Recent studies indicate that bone-targeted nanocarriers loaded with the Piezo1 activator Yoda1 can activate Piezo1 in defect sites of osteoporotic mice. This activation enhances osteogenic and angiogenic coupling via the Piezo1-YAP/β-catenin axis, accelerating bone remodeling in defect areas.

Building on this background, we hypothesize that the mechanical loading provided by the CDC technique accelerates bone regeneration by activating the Piezo1-YAP-β-catenin signaling axis, thereby enhancing osteogenic-angiogenic coupling. This study aims to elucidate this mechanism, providing a theoretical basis for the optimization of mechanical protocols in distraction osteogenesis.

## Materials and methods

### Animal acquisition and husbandry

Adult male Sprague-Dawley rats were procured from the Southwest Medical University Laboratory Animal Center [License No. SCXK (Chuan) 2024-0046]. The animals were maintained in a specific pathogen-free (SPF) facility with strictly controlled temperature and a standard light-dark cycle. Prior to surgical intervention, the rats underwent a 7-day acclimatization period with *ad libitum* access to standard rodent chow and sterile water. All experimental protocols strictly adhered to the Guidelines for the Care and Use of Laboratory Animals issued by the Affiliated Hospital of Southwest Medical University. Ethical approval for this study was granted by the institutional Animal Ethics Committee (Approval No. 20250930-008).

### Surgical protocol and post-operative management

To minimize variability, all surgical procedures were performed by a single experienced surgical team on a cohort of 60 rats. Anesthesia was induced via an intraperitoneal injection of 2% sodium pentobarbital (30 mg/kg), supplemented with preoperative prophylactic penicillin. Under aseptic conditions, a custom-fabricated unilateral external fixator (School of Mechanical Engineering, Xinjiang University) was secured to the right femur using four stainless steel self-tapping screws, followed by a mid-diaphyseal transverse osteotomy.

Post-operatively, pin sites were cleansed daily with an antibiotic solution. Infection prophylaxis consisted of intramuscular penicillin injections for three consecutive days post-surgery. Animals were individually housed to facilitate recovery, with unrestricted access to food and water.

### Distraction osteogenesis and CDC regimen

**FIGURE 1:**
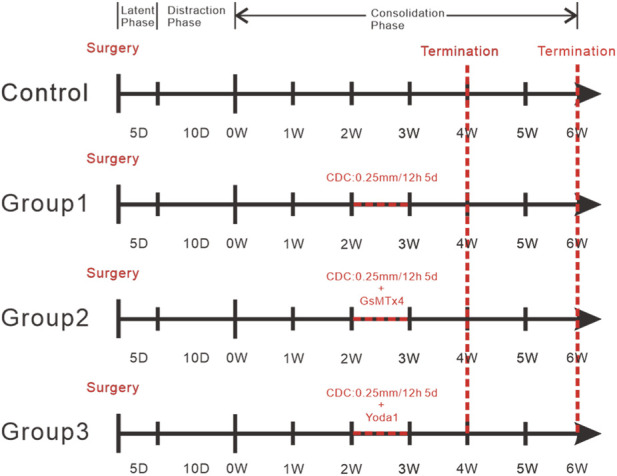
The schematic diagram of the cyclic distraction–compression (CDC) protocol. The CDC technique was performed in the middle phase of bone consolidation (started at week three in the consolidation period). Control, no any interventions; Group1, 2.5-day compression and 2.5-day distraction at a rate of 0.25 mm/12 h for 5 days; Group 2, 2.5-day compression and 2.5-day distraction at a rate of 0.25 mm/12 h for 5 days, with daily local injection of 100 μL GsMTx4 (mechanosensitive ion channel inhibitor) at 48 μmol/L; Group 3, 2.5-day compression and 2.5-day distraction at a rate of 0.25 mm/12 h for 5 days, with daily local injection of 100 μL of Yoda1 (pharmacological activator of Piezo1) at a concentration of 100 μmol/L.

Following a 5-day latency period, distraction was initiated at a rate of 0.25 mm/12 h for 10 days, yielding a cumulative distraction gap of 5.0 mm. Upon completion of distraction, the animals entered a 6-week consolidation phase. Commencing at week three of the consolidation period (mid-healing phase), rats were randomized into four groups to undergo the specified Cyclic Distraction–Compression (CDC) protocol: Control Group (n = 15): Received no additional intervention. Group 1 (n = 15): Subjected to a dynamic loading protocol consisting of 2.5 days of compression followed by 2.5 days of distraction (0.25 mm/12 h) over a 5-day duration. Group 2 (n = 15): Subjected to the Group 1 loading protocol, with concurrent daily local injections of 100 μL GsMTx4 (Piezo1 selective inhibitor) at a concentration of 48 μmol/L. Group 3 (n = 15): Subjected to the Group 1 loading protocol, with concurrent daily local injections of 100 μL Yoda1 (Piezo1 selective agonist) at a concentration of 100 μmol/L (Figure 1).

Yoda1 (a pharmacological activator of Piezo1) and GsMTx4 (a mechanosensitive ion channel inhibitor) were administered via local injection into the distraction gap. The concentrations of Yoda1 (100 μmol/L) and GsMTx4 (48 μmol/L), as well as the injection volume (100 μL per day), were selected based on previously published studies and preliminary experiments to achieve effective local modulation of mechanosensitive signaling while minimizing tissue disruption.

Both agents were freshly prepared prior to injection using DMSO as solvent and 5% ethanol as diluent to ensure consistency across treatments.

This local administration strategy was designed to limit systemic exposure and primarily target the distraction regenerate microenvironment.

Rats were euthanized after the 4-week and 6-week consolidation periods via an intraperitoneal overdose of sodium pentobarbital (150 mg/kg). Bilateral femurs were then harvested for subsequent analysis.

At the 4-week time point, n = 6 specimens per group were used for histological and immunohistochemical analyses. At the 6-week time point, n = 9 specimens per group were allocated to histological (n = 3), biomechanical testing (n = 3), and micro-CT/immunohistochemical analyses (n = 3).

### Radiographic assessment

Longitudinal anteroposterior (AP) X-ray evaluation of the distraction zone was conducted weekly after brief inhalation anesthesia with 2% isoflurane. A digital radiographic system (HF400VA, MIKASA X-RAY Co., Ltd., Tokyo, Japan) was utilized with consistent acquisition parameters (44 kV, 4.5 mA) throughout the study.

### Micro-computed tomography (Micro-CT) analysis

At the 6-week endpoint, the micro-architecture of the regenerate bone was quantitatively assessed using a micro-CT system (SkyScan 1176, Bruker, Rheinstetten, Germany; n = 3 per group). Scanning was performed using an Al + Cu filter at 80 kV and 313 μA, with a voxel size of 0.9 μm and an exposure time of 0.203 s. Images were reconstructed using NRecon software (SkyScan), and 3D morphometric analysis was conducted using CTAn software (SkyScan) in accordance with manufacturer guidelines. The region of interest (ROI) was defined as the distraction zone delineated by the periosteal boundary between the proximal and distal osteotomy ends (Perrien et al., 2012). Bone mineral density (BMD) and bone volume fraction (BV/TV) were quantified strictly within the osseous tissue of the ROI. The sample size was determined based on previous similar *in vivo* studies and practical constraints associated with animal experiments.

### Biomechanical characterization

Mechanical properties were evaluated via three-point bending tests using a materials testing machine (RGM-3005T, ShenZhen Reger Instrument Co., Ltd., China) within 24 h of harvest (n = 3 per group, 6-week time point). External fixators and residual soft tissues were removed, and the contralateral intact femurs served as internal controls. Specimens were positioned with the long axis perpendicular to the supports (18 mm span). A load was applied to the distraction callus in the AP direction at a displacement rate of 0.5 mm/min until failure. The ultimate load, elastic modulus (E-modulus), failure energy, and stiffness were derived from the load-displacement curves using the system’s software (REGER) and normalized to the contralateral femur.

### Histological and immunohistochemical analysis

Specimens were fixed in 10% neutral buffered formalin for 48 h and stored in 75% ethanol. At each designated time point, three randomly selected specimens per group were dehydrated via graded alcohols, defatted in xylene, and embedded in methyl methacrylate. Undecalcified sections (10 μm) were prepared using a heavy-duty microtome (HistoCore AUTOCUT, Leica, Wetzlar, Germany) and stained with Von Kossa, Masson Trichrome, Goldner Trichrome, and Safranin O for histomorphometry.

For immunohistochemistry (IHC), the remaining three specimens per group were decalcified in 10% EDTA for 4 weeks, dehydrated, cleared, and paraffin-embedded. Sections (5 μm) were obtained using a rotary microtome (RM2135, Leica). Following deparaffinization and rehydration, endogenous peroxidase activity was quenched with 0.3% hydrogen peroxide for 20 min. Antigen retrieval was performed using 0.4% pepsin at 37 °C for 25 min, followed by blocking with 5% goat serum at 37 °C for 30 min. Sections were incubated overnight at 4 °C with primary antibodies against Piezo1 (1:200, Proteintech), YAP (1:200, CST), β-catenin (1:100, CST), VEGF (1:100, Santa Cruz), CD31 (1:50, Abcam), RUNX2 (1:100, Santa Cruz), and OPN (1:100, ProteinTech). Detection was achieved using a secondary antibody (PV6000, ZSGB-BIO) incubated at 37 °C for 1 h, followed by a streptavidin-horseradish peroxidase system (ZLI-9019, ZSGB-BIO) and hematoxylin counterstaining. Negative control sections were processed in parallel by omitting the primary antibody, while all other procedures were kept identical, to verify staining specificity. For immunohistochemical quantification, three non-overlapping sections were analyzed per specimen, and three random fields within the regenerate zone were captured per section at ×200 magnification. Field selection was performed in a randomized manner within the defined region of interest to minimize selection bias. Quantitative analysis was conducted using Image-Pro Plus 6.0 software (Media Cybernetics, United States). A consistent threshold was applied across all images to determine the positive staining area, and the mean value per specimen was calculated and used as one biological replicate for statistical analysis. A total of three biological replicates (n = 3) were included per group for immunohistochemical quantification. All image acquisition and quantitative analyses were performed by investigators blinded to group allocation.

### Statistical analysis

Statistical analysis was performed using SPSS version 27.0 (SPSS Inc., Chicago, IL, United States) and GraphPad Prism v.6.0 (GraphPad Inc., San Diego, CA, United States). Data are presented as mean ± standard deviation (SD). Normality of data distribution was assessed using the Shapiro–Wilk test.For comparisons among multiple groups, one-way analysis of variance (ANOVA) followed by Tukey’s post hoc multiple comparisons test was used when data were normally distributed. For non-normally distributed data, the Kruskal–Wallis test followed by Dunn’s multiple comparisons test was applied. A two-sided p < 0.05 was considered statistically significant. Where applicable, exact p-values are reported.

## Results

### Radiographic assessment of bone regeneration

**FIGURE 2:**
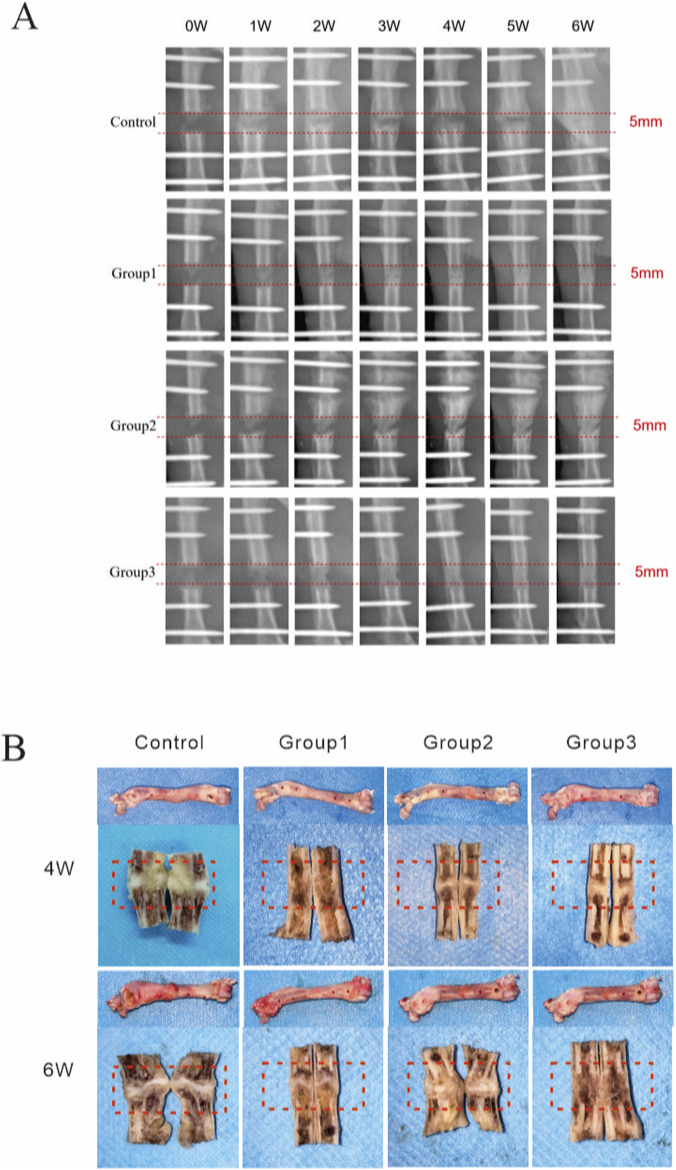
Different patterns of the CDC technique affect bone regeneration during distraction osteogenesis (DO). **(A)** X-ray images of the distraction regenerate weekly until a 6-week consolidation duration is terminated. **(B)** The general image of specimens after 2, 4, and 6 weeks of consolidation.

Sequential digital radiography tracked the evolution of the regenerate bone throughout the consolidation phase. During the initial 2 weeks, no discernible disparities in bone formation were evident across the four experimental groups (Figure 2A).

**FIGURE 3:**
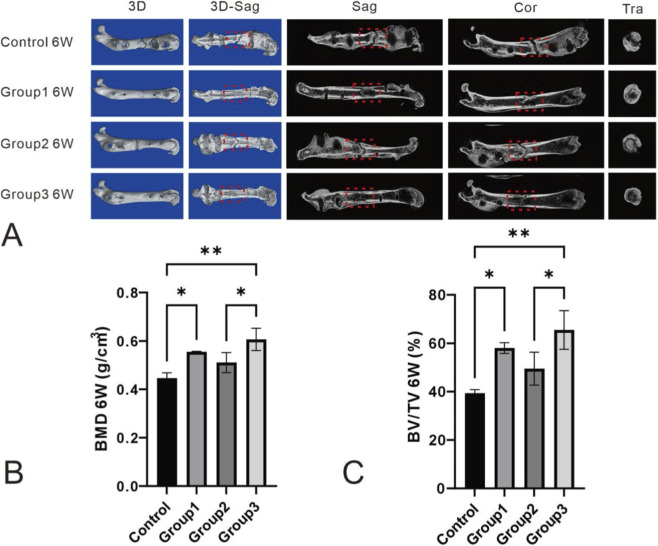
Results of micro-CT evaluation demonstrating promotion in regenerate quality after the CDC technique (n = 3 biological replicates per group). **(A)** Representative 3D micro-CT images of the distraction zone at the termination of the 6-week consolidation. **(B,C)** Quantitative evaluation of BMD and BV/TV, manifesting that the two values in Group1 and Group3 were significantly higher than those in Control and Group1 (*:*p* < 0.05, **:*p* < 0.01).

However, following the implementation of the CDC protocol at week 3, a marked acceleration in bone regeneration volume was observed in Groups 1 and 3. By the conclusion of the 6-week consolidation period, radiographic imaging revealed persistent radiolucent gaps between the proximal and distal osteotomy ends in both the Control group and Group 2. In contrast, Groups 1 and 3 achieved osseous bridging. Furthermore, compared to Group 2, the callus volume and structural continuity were substantially superior in Groups 1 and 3. These radiographic findings were corroborated by gross examination of the dissected femora (Figure 2B) and micro-CT reconstruction (Figure 3A), which confirmed that primary recanalization of the medullary cavity was successfully established in Groups 1 and 3.

### Micro-architectural analysis via Micro-CT

Quantitative micro-CT analysis at the 6-week endpoint highlighted distinct structural differences. Representative reconstructions showed that Groups 1 and 3 possessed well-preserved medullary continuity and evidence of advanced remodeling, whereas the distraction zones in the Control group and Group 2 displayed incomplete bridging (Figure 3A).

Statistically, significant variations were noted in bone mineral density (BMD). Specifically, BMD values in Group 1 (0.5559 ± 0.0017 g/cm^3^) and Group 3 (0.6071 ± 0.0463 g/cm^3^) were significantly elevated compared to the Control group (0.4461 ± 0.0223 g/cm^3^; p < 0.05). Moreover, Group 3 exhibited a significantly higher BMD than Group 2 (0.5109 ± 0.0418 g/cm^3^; p < 0.05) (Figure 3B).

Regarding the bone volume fraction (BV/TV), both Group 1 (58.3574% ± 3.5672%) and Group 3 (64.853% ± 8.6337%) demonstrated significantly superior values relative to the Control group (40.0123% ± 1.9884%; p < 0.05). Additionally, the BV/TV in Group 3 was significantly greater than that of Group 2 (49.3258% ± 8.3317%; p < 0.05) (Figure 3C). Collectively, these data suggest that the CDC protocol augments osteogenic capacity during distraction osteogenesis, potentially through the potentiation of Piezo1-mediated mechanotransduction.

### Biomechanical characterization

**FIGURE 4:**
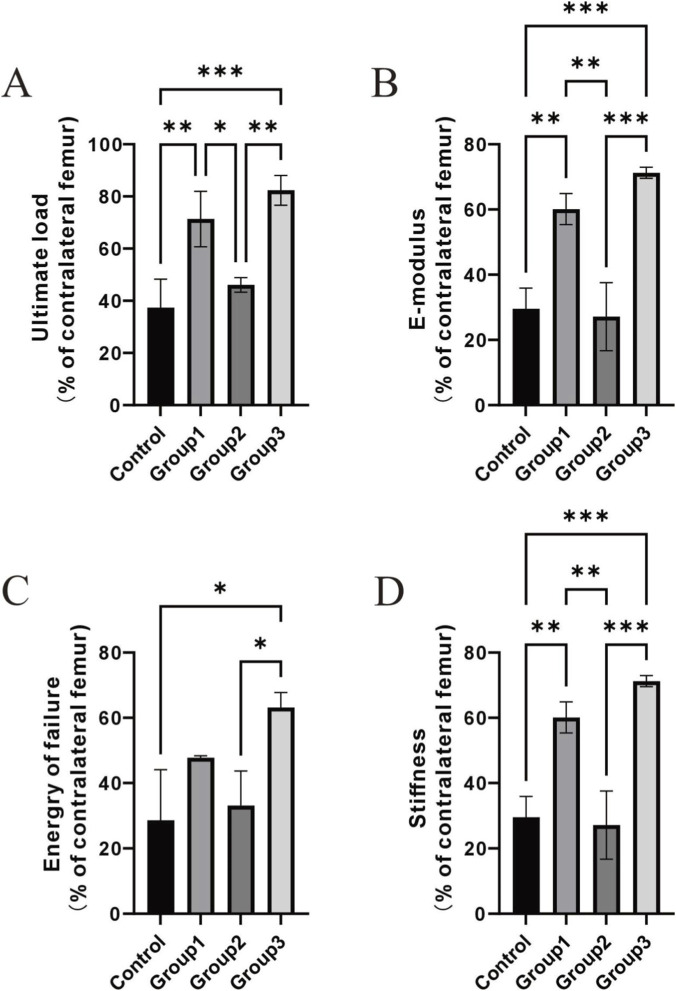
Results of biomechanical properties (n = 3 biological replicates per group). **(A)** Ultimate load. **(B)** Elastic modulus (E-modulus). **(C)** energy of failure. **(D)**. stiffness (*:*p* < 0.05, **:*p* < 0.01, ***:*p* < 0.001).

Functional mechanical competency of the regenerate bone was assessed via three-point bending tests after 6 weeks of consolidation. Both Group 1 and Group 3 displayed superior biomechanical performance compared to the Control group. Conversely, no statistically significant difference was detected between the Control group and Group 2 (Figure 4).

### Histological observations

**FIGURE 5:**
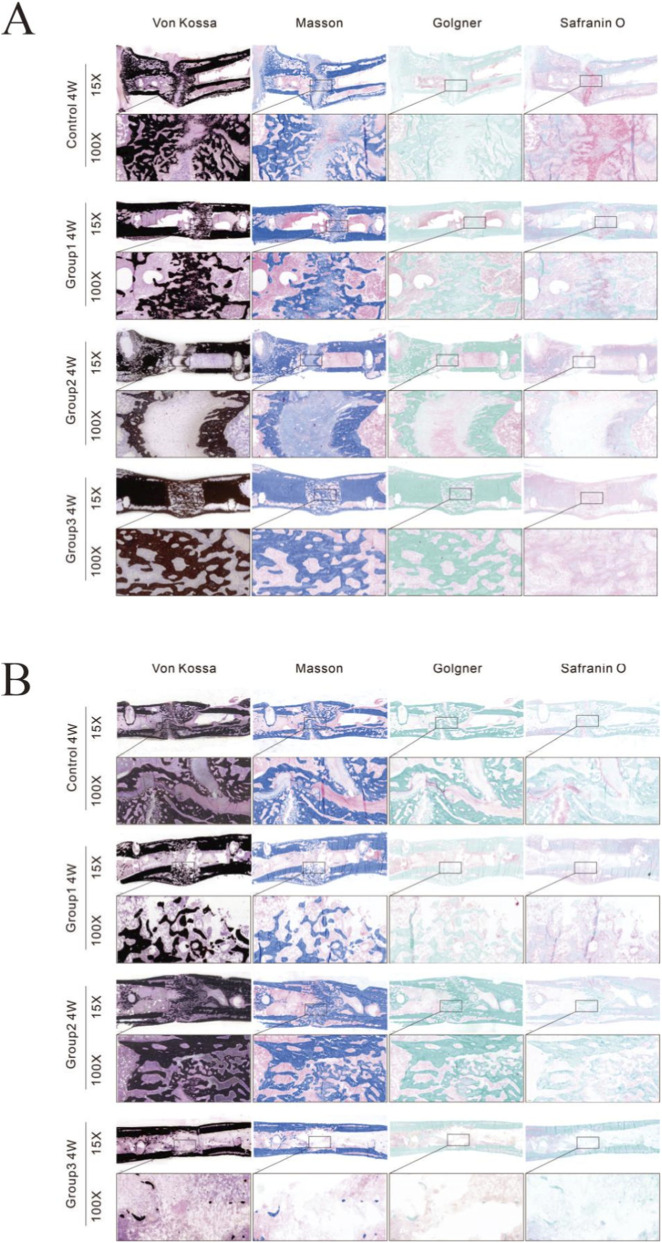
Histomorphological analysis of bone regeneration during the consolidation period (n = 3 biological replicates per group). **(A)** Staining results of each group at 4 weeks; **(B)** Staining results of each group at 6 weeks. These results demonstrate enhanced bone formation and mineralization in Group 1 and Group 3 compared with the Control group and Group 2.

The histomorphological characteristics of undecalcified sections were evaluated using Von Kossa, Masson Trichrome, Goldner Trichrome, and Safranin O staining. After 4 weeks of consolidation, Von Kossa staining depicted persistent radiolucent discontinuities within the distraction gap of the Control group and Group 2. In contrast, Groups 1 and 3 exhibited robust new bone formation characterized by larger volumes and superior quality (Figure 5A).

At the 6-week terminal point, while narrow gaps remained discernible in the Control and Group 2 specimens, complete bony union was evident in Groups 1 and 3. Furthermore, the medullary canals in the latter groups had undergone extensive remodeling, restoring patency. These findings were consistently supported by Masson Trichrome and Goldner Trichrome staining, which confirmed accelerated osteogenesis in Groups 1 and 3. Safranin O staining revealed the presence of prominent chondrocytic clusters within the center of the distraction zone in the Control group and Group 2 at week 6, indicative of delayed endochondral ossification and incompletely mineralized regenerate bone (Figure 5B).

### Immunohistochemical profiling

Immunohistochemical analysis elucidated the molecular response to the CDC intervention. At week 4, Group one showed upregulated expression of Piezo1, YAP, β-catenin, CD31, VEGF, RUNX2, and OPN relative to the Control and Group 2. Statistical analysis confirmed that β-catenin and OPN levels were significantly higher than those in the Control and Group 2 (p < 0.05), while YAP expression significantly exceeded the Control (p < 0.05), and Piezo1 expression was significantly elevated compared to Group 2 (p < 0.05). Notably, Group 3 demonstrated a robust and statistically significant upregulation of all assayed markers (Piezo1, YAP, β-catenin, CD31, VEGF, RUNX2, and OPN) compared to both the Control group and Group 2 (p < 0.05). Furthermore, expression intensities in Group 3 surpassed those observed in Group 1.

**FIGURE 6:**
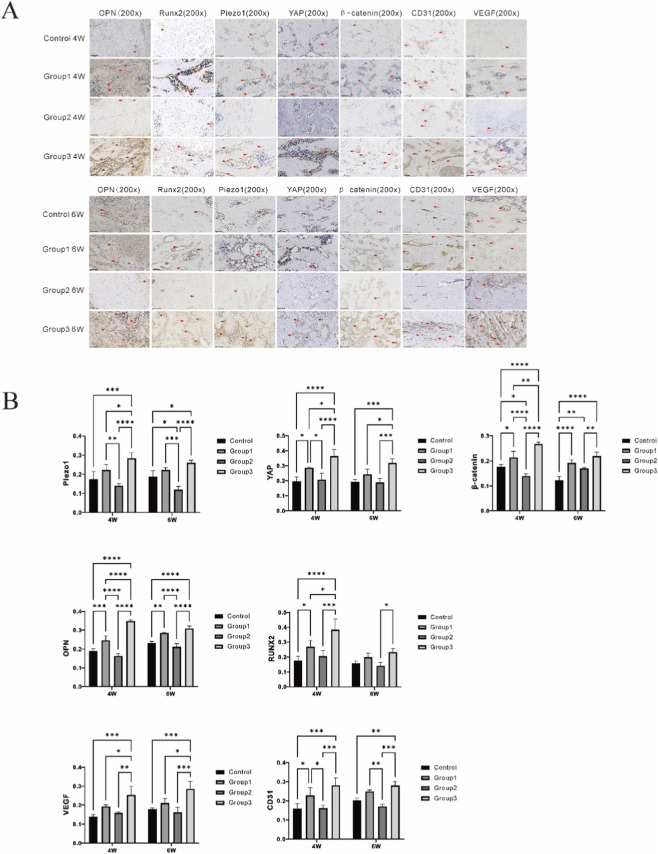
Immunohistochemical analysis of Piezo1, YAP, β-catenin, CD31, VEGF, RUNX2, and OPN. (n = 3 biological replicates per group). **(A)** Representative images at 4 weeks and 6 weeks. **(B)** Semi-quantitative analysis at 4 weeks and 6 weeks. (*:*p* < 0.05, **:*p* < 0.01, ***:*p* < 0.001, ****:*p* < 0.0001).

At the 6-week interval, Group 1 sustained elevated expression levels of the aforementioned markers compared to the Control and Group 2; specifically, β-catenin, CD31, and OPN were significantly upregulated (p < 0.05). Consistent with week four findings, Group 3 exhibited significantly higher expression levels of Piezo1, YAP, β-catenin, CD31, VEGF, RUNX2, and OPN compared to both the Control group and Group 2 (p < 0.05) (Figure 6).

## Discussion

Distraction osteogenesis (DO) remains a powerful reconstructive strategy because it leverages endogenous tissue regeneration under controlled mechanical conditions, yet its clinical translation is frequently limited by slow regenerate maturation and a prolonged consolidation period that increases frame-related complications and patient burden. The cyclic distraction–compression (CDC) maneuver (also termed the accordion technique) has been proposed as a drug-free, low-cost, and clinically feasible approach to enhance regenerate quality; however, its efficacy appears to be protocol- and timing-dependent, and its molecular mechanism has remained insufficiently defined. In the present work, we applied CDC during the mid-consolidation phase and provide evidence that CDC accelerates regenerate mineralization and remodeling in association with a mechanotransduction program involving Piezo1–YAP–β-catenin signaling and the associated osteogenic–angiogenic coupling.

Our *in vivo* data demonstrate a consistent pro-regenerative effect of CDC when initiated at week three of consolidation: radiographs and gross inspection showed that bony bridging and medullary recanalization were achieved in the CDC group (Group 1) and were most pronounced in the Piezo1 agonist–augmented CDC group (Group 3), whereas gaps persisted in the control group and the Piezo1 inhibitor–treated CDC group (Group 2). This qualitative improvement was corroborated by micro-CT, where both BMD and BV/TV increased significantly with CDC, and were further enhanced by Yoda1 (Group 3), while GsMTx4 blunted these gains. Importantly, biomechanical testing aligned with imaging outcomes: mechanical performance improved in Groups 1 and 3 but not significantly in Group 2, suggesting that Piezo1 activity is not merely a marker but a functional determinant of CDC-induced strengthening. Histology provided complementary insight, showing persistent gaps and delayed maturation (including prominent cartilage in the distraction zone) in control and Group 2, whereas Groups 1 and 3 exhibited larger volumes of mineralized tissue and more complete medullary restoration. Together, these findings indicate that CDC improves both the quantity and quality of regenerate bone, and that pharmacologic augmentation of Piezo1 amplifies this effect, while Piezo1 inhibition compromises it. Notably, the consistency of findings across independent evaluation modalities (radiography, micro-CT, histology, and biomechanical testing) strengthens the reliability of these observations despite the relatively limited sample size in certain analyses.

Clinical and preclinical studies of the accordion technique have reported mixed results, likely reflecting differences in the timing of application, the magnitude and rate of compression/distraction cycles, and the maturity of the nascent vascular network. A recent mouse study systematically testing three time points concluded that the accordion technique did not improve healing under their parameters and suggested that early consolidation application may damage newly formed vascular tissue (David et al., 2024). These observations underscore that CDC is not universally beneficial; rather, it may require a mechanobiological “window” when the regenerate has sufficient structural integrity and vascular organization to tolerate controlled micromotion yet remains responsive to pro-osteogenic cues. Our protocol initiated CDC at week three of consolidation—a phase when the regenerate has typically transitioned from a highly fragile fibrovascular interzone toward more mineralized trabecular structures—thereby plausibly avoiding destructive overloading of immature vessels while still leveraging mechanosensitive pathways. Consistent with this concept, prior work in a rat femoral DO model showed that CDC during mid-consolidation improved regenerate volume, continuity, and mechanical properties and increased angiogenic/osteogenic signaling (including HIF-1α and VEGF), with better outcomes at moderately increased amplitude or slower rate (Yanshi et al., 2022b). Taken together, these findings suggest that protocol optimization (timing, amplitude, rate, and cycle number) is central to extracting reproducible benefit from CDC. Notably, the CDC protocol used in this study (2.5 days of compression followed by 2.5 days of distraction at 0.25 mm/12 h during mid-consolidation) represents a defined and timing-specific regimen, which may provide an optimized mechanical environment for Piezo1-mediated mechanotransduction.

Mechanistically, our study connects CDC efficacy to Piezo1 activation. Piezo1 is an established mechanosensitive cation channel in bone, and its genetic loss in osteoblast lineage cells impairs bone formation and compromises bone strength, supporting a causal role in load-dependent osteogenesis (Weijia et al., 2019). Beyond its role in baseline homeostasis, Piezo1/2 can integrate mechanical cues into a broader transcriptional response by promoting Ca^2+^ influx and downstream activation of calcineurin-dependent transcription factors, including YAP1 and β-catenin, via concerted dephosphorylation and complex formation (Taifeng et al., 2020). In our model, immunohistochemistry revealed that CDC increased Piezo1, YAP, and β-catenin expression in the regenerate, accompanied by upregulation of osteogenic markers (RUNX2 and OPN); these effects were strongest with Yoda1 and weakest with GsMTx4. This pattern is consistent with a working model in which CDC-induced mechanical strain may activate Piezo1, thereby contributing to YAP- and β-catenin–associated signaling involved in osteoblast differentiation and matrix mineralization.

These observations are also coherent with broader principles of mechanotransduction. YAP/TAZ are canonical mechanosensitive transcriptional coactivators that respond to matrix stiffness and cytoskeletal tension, enabling cells to translate physical forces into gene expression changes (Sirio et al., 2011). Meanwhile, Wnt/β-catenin signaling is a central axis for osteoblast development and bone mass regulation, and its integration with mechanically regulated pathways has been increasingly appreciated (Roland and Michaela, 2013). Our data do not prove direct molecular binding events within the regenerate niche, but the coordinated elevation of Piezo1, YAP, and β-catenin—together with functional sensitivity to Piezo1 agonism/inhibition—strongly suggests that CDC acts upstream of a Piezo1-driven transcriptional network that favors intramembranous ossification and accelerated remodeling. Therefore, our findings should be interpreted as supporting an association between Piezo1 activity and YAP/β-catenin signaling, rather than establishing a definitive causal signaling cascade.

Importantly, Piezo1 is expressed in multiple mechanoresponsive cell types within the distraction regenerate, including osteoblast-lineage cells, endothelial cells, and stromal progenitors. Therefore, the effects observed in this study likely represent the integrated response of multiple cellular compartments rather than a single cell-type-specific mechanism. The current pharmacological approach does not allow us to distinguish the relative contribution of these cell populations.

A defining feature of successful DO is the tight coupling between angiogenesis and osteogenesis, as vessels provide oxygen, nutrients, osteoprogenitor trafficking routes, and paracrine cues that shape ossification patterns. A specialized endothelial subtype has been shown to couple angiogenesis to osteogenesis in skeletal development and repair, emphasizing that vascular quality—not only vessel density—matters for bone formation (Anjali et al., 2014). However, the present study did not distinguish endothelial subtypes or assess vascular perfusion, which represents a limitation. In our study, CDC increased the expression of the angiogenic markers VEGF and CD31 within the regenerate, again with maximal induction in the Piezo1 agonist group and attenuation with Piezo1 inhibition. These findings support the interpretation that CDC does not solely stimulate osteoblast differentiation but also promotes a pro-angiogenic microenvironment, thereby enabling faster mineralization and structural maturation. Given the known expression of Piezo1 in both endothelial and osteogenic compartments, it is possible that Piezo1 coordinates angiogenesis–osteogenesis coupling through multi-lineage mechanotransduction mechanisms.

Notably, previous work suggested that CDC may trigger tissue hypoxia and activate HIF-dependent pathways (with increased HIF-1α and VEGF), promoting osteogenic–angiogenic coupling during consolidation (Yanshi et al., 2022b). Our results are consistent with this framework at the level of VEGF and endothelial markers, but extend it by positioning Piezo1 as a mechanosensory “entry point” for CDC-induced signaling. Because Piezo1 is expressed in multiple mechanoresponsive cell types, including osteoblast lineage cells and endothelial cells, it may coordinate both osteogenic differentiation and vascular adaptation under cyclic loading. Future mechanistic studies will be required to dissect the relative contribution of Piezo1 activation in osteoblasts versus endothelial compartments and to determine how Piezo1–YAP–β-catenin signaling interfaces with hypoxia/HIF and Notch-dependent vascular programs during DO.

From a translational perspective, CDC remains attractive because it can be implemented through existing external fixator systems without adding graft-related morbidity, complex biologics, or high-cost cell products. Protocol sensitivity highlighted by recent negative or neutral studies also implies that clinical adoption should be accompanied by careful parameterization and objective monitoring (e.g., serial micro-CT in preclinical optimization, or standardized radiographic scoring and dynamization thresholds clinically). Our data suggest an additional layer of control: Piezo1 modulation may pharmacologically tune the regenerate’s responsiveness to mechanical stimulation. Yoda1 was identified as a small-molecule agonist capable of chemically activating Piezo1, providing proof-of-concept that Piezo1 is druggable (Ruhma et al., 2015). Conversely, GsMTx4 has been reported to inhibit Piezo1 activity, supporting its utility as a mechanistic probe and acknowledging potential off-target effects typical of mechanosensitive channel modulators) (Gunasekaran and Badri, 2013b). Although direct clinical translation of these agents would require extensive safety evaluation—given Piezo1’s functions across multiple organs—our findings motivate the broader idea of combining optimized mechanical regimens with targeted mechanotransduction modulation to shorten consolidation time and improve regenerate quality.

This study has several limitations that should be considered when interpreting the results. First, although pharmacological modulation using Yoda1 and GsMTx4 supports the involvement of mechanosensitive signaling, these agents do not provide absolute specificity. In particular, GsMTx4 can affect multiple mechanosensitive ion channels, and therefore the observed effects should be interpreted as modulation of mechanotransduction rather than exclusively Piezo1-specific activity. In addition, formal pharmacokinetic and dose–response analyses were not performed, and a dedicated vehicle control group was not included, which may limit the ability to fully exclude potential injection- or solvent-related effects. Second, although pharmacologic activation and inhibition support the involvement of Piezo1, genetic approaches (e.g., conditional Piezo1 deletion in specific cell populations) are required to establish cell-type-specific causality and exclude off-target effects. Moreover, because Piezo1 is expressed in multiple cell types within the regenerate microenvironment, the present study cannot determine the relative contribution of osteoblast-lineage cells, endothelial cells, or other stromal populations. Third, the sample size for micro-CT and biomechanical analyses was relatively small (n = 3 per group), which may limit statistical power. Although consistent trends were observed across multiple independent evaluation modalities, validation in larger cohorts will be necessary. Fourth, pathway activity was primarily assessed by immunohistochemistry. Although negative controls were performed, representative images were not included, and subcellular localization of YAP and β-catenin was not evaluated. Given that nuclear translocation is a key indicator of pathway activation, future studies using immunofluorescence or high-resolution imaging will be required. Fifth, only a single CDC protocol and drug dosing regimen were tested. Systematic optimization of mechanical parameters (e.g., amplitude, rate, cycle number, and timing) and pharmacological dosing will be necessary to define the most effective treatment strategy. Finally, long-term outcomes and validation in larger animal models are required before clinical translation. Future studies incorporating genetic models, pathway-specific inhibition, and quantitative assessment of nuclear signaling activity will be essential to validate the direct involvement and hierarchical structure of this signaling axis.

## Conclusion

In summary, our study demonstrates that CDC applied during mid-consolidation improves regenerate mineralization, structural remodeling, and mechanical competence, and that these benefits are potentiated by Piezo1 activation while being blunted by Piezo1 inhibition. The coordinated induction of Piezo1, YAP, and β-catenin signaling together with upregulation of osteogenic and angiogenic markers supports a model in which Piezo1–YAP–β-catenin–associated signaling may act as an important mechanotransduction pathway linking cyclic mechanical stimulation to osteogenic–angiogenic coupling during DO. This mechanistic framework provides a rational basis for optimizing CDC protocols and for exploring mechanosensory-targeted adjuvant strategies aimed at shortening consolidation and improving clinical outcomes.

## Data Availability

The original contributions presented in the study are included in the article/supplementary material, further inquiries can be directed to the corresponding authors.
